# A novel *GJA8* mutation causing a recessive triangular cataract

**Published:** 2008-05-09

**Authors:** Werner Schmidt, Norman Klopp, Thomas Illig, Jochen Graw

**Affiliations:** 1 Center of Ophthalmology, Universities of Gießen and Marburg, Gießen, Germany; 2 Institute of Epidemiology, Helmholtz Center Munich - German Research Center for Environmental Health, Neuherberg, Germany; 3 Institute of Developmental Genetics, Helmholtz Center Munich - German Research Center for Environmental Health, Neuherberg, Germany

## Abstract

**Purpose:**

The aim of the study was to characterize the underlying mutation in a consanguineous family having cataracts.

**Methods:**

Family D having congenital cataracts was treated at the University Eye Clinics at Giessen (Germany). Lens material from surgeries was collected, immediately frozen at –80 °C, and used for cDNA production. Blood was taken from the proband and available family members. Polymerase chain reaction (PCR)-amplified DNA fragments were characterized by sequencing and restriction digestion.

**Results:**

The proband, AD, has a dense, triangular nuclear cataract. The parents are consanguineous, and the mother and grandmother suffer from a discrete, symmetric opacity of the fetal lens nucleus. The proband’s lens cDNA showed a homozygous insertion of one G after position 776 of the *GJA8* gene, leading to a frame shift and 123 novel amino acids. The homozygous mutation was confirmed in the genomic DNA and is also present in the cataract-operated brother of the proband; all other family members investigated were heterozygous. The mutation could not be detected in 96 healthy controls from Germany.

**Conclusions:**

The ins776G mutation most likely causes a recessive triangular cataract with variable expressivity of a weak phenotype in heterozygotes.

## Introduction

A broad variety of cataract mutations have been characterized in man and mouse within the past 10 years. The analysis of congenital (mainly dominant) cataracts discovered an unexpectedly high number of mutations in genes coding for γ-crystallins and to a less extent in those genes coding for α- and β-crystallins. Furthermore, structural proteins in the lens represent those forming the beaded filaments or membrane proteins. Among them, the most prominent ones are the main intrinsic protein (MIP), which belongs to the family of aquaporins, and gap-junction proteins, which belong to the connexin families. Mutations in the corresponding genes *MIP*/*Mip*, *GJA3*/*Gja3*, and *GJA8*/*Gja8* (encoding the gap junction membrane channel protein α3 and α8, respectively) have been shown to underlie some congenital, mainly dominant cataracts in mouse and man (for a review see [1]). Additional causes for congenital, hereditary cataracts are mutations affecting enzymes of sugar metabolism (e.g., *GALK1* [2]) or other metabolic disorders like hyperferritinemia [3].

Whereas recessive mutations in genes leading to metabolic disorders are frequently observed, recessive mutations affecting structural lens proteins are rather rare. Nevertheless, in recent years an increasing number of reports has been published describing recessive mutations in *BFSP1* [4], *CRYAA* [5,6], *CRYBB1* [7], *CRYBB3* [8], *GJA8* [9], *GCNT2* [10], *HSF4* [11,12], and *LIM2* [13].

Even this short overview indicates a broad heterogeneity not only at the clinical side but also at the genetic side. There is no clear genotype-phenotype correlation, and mutations affecting the same genes might act in a recessive and dominant manner. Here, we describe three generations of a consanguineous family. In its third generation, a unique form of triangular nuclear cataract was observed. The molecular reason is most likely a single base-pair insertion in the *GJA8* gene. The mutation in this gene is mainly associated with dominant cataracts.

## Methods

The study adopted the tenets of the Declaration of Helsinki as family members were informed about the study, its outcome, and their role before seeking consent as per standard norms. The study was also approved by the Institutional Ethical Committee of the University of Gießen, Gießen, Germany. Clinical details including the family’s health history were recorded at the Center of Ophthalmology at the University of Gießen. A senior pediatric ophthalmologist (W.S.) operated on the proband. The parents and other family members were examined with a slit-lamp (Zeiss, Oberkochen, Germany); pictures were taken by an operation microscope and coaxial illumination.

**Table 1:** Primers used for polymerase chain reaciton.

**Gene**	**Laboratory number**	**Sequence (5′->3′)**	**T_m_ [°C]; fragment size (bp)**
*CRYAA* (L1)	62065	TGCGCTGCCCAGAGGC	50–61 ~700
*CRYAA* (R1)	62066	CACCCTGAGACCCTAAGCTCTCC	
*CRYAB* (L1)	62068	CCCCTCACACTCACCTAGCCAC	50–61 ~690
*CRYAB* (R1)	62067	CTTGATAATTTGGGCCTGCCC	
*CRYBB2* (L1)	MWG	CAGGGGCTGGTGTCACTGGTC	52–63 ~600
*CRYBB2* (R1)	MWG	TGGAGGGTTCCTGGGCAGC	
*CRYGA* (L2)	64912	CACCCCTCTGTCAACAACC	50–61 ~490, 550, 600, 680
*CRYGA* (R1)	61952	ATCACAACAAGGCAGGCAC	
*CRYGB* (L1)	61954	AGATTTTAAAGGAGAAAAGTGGAAAAC	45–56 ~580
*CRYGB* (R1)	61955	ACAGCAACCAGAAAACATCTGC	
*CRYGC* (L1)	61957	TTTGTGTTGTTCTTGCCAACGC	50–61 ~680
*CRYGC* (R1)	61956	CAAAATGGGAAATTGGTAGTGTTAAGC	
*CRYGD* (L1)	61958	CCACCAGCTCAGCACCGC	50–61 ~580
*CRYGD* (R1)	61959	CCAAATTAAGAAACAACAAAGGAGGAC	
*CRYGS* (L1)	62073	CCATTCCTGAATTTCTTTCAGCAC	50–61 ~680
*CRYGS* (R1)	62074	TGGACCACAAGGCCAGCC	
*GJA3* (L2)	Invitrogen	ATGGGCGACTGGAGCTTTC	60–65 ~680
*GJA3* (R2)	Invitrogen	ATCTCCAGCATGTTGAGCAGC	
*GJA3* (L3)	Invitrogen	TGGACTGCTTCATCTGCCAGGCC	61–63 ~700
*GJA3* (R3)	Invitrogen	CTAGATGGCCAAGTCCTCCGGTC	
*GJA8* (L3)	Invitrogen	CAGCCGGTGGCCCTGC	51–62 ~700
*GJA8* (R3)	Invitrogen	TCACTTCATACGGTTAGATCGTCTGAC	
*LIM2* (L1)	MWG	CTCGGGCAGGCTCTGCCAC	57–64 ~250
*LIM2* (R2)	64918	CGGAGATGGCGCATAGGGC	
*MIP* (L2)	64915	CCCAGGCACTGTGACCATCC	50–61 ~700
*MIP* (R1)	62072	CTTTCTTCATCTAGGGGCTGGC	

Primers used for amplification of lens cDNA of various genes by polymerase chain reaction (PCR).

The lens material after cataract surgery was frozen immediately on dry ice and kept at –80 °C for a few days. RNA samples from lenses were reverse transcribed to cDNA using the Ready-to-Go kit (GE Health Care, Freiburg, Germany). Reverse transcription polymerase chain reaction (RT–PCR) using cDNA as template was performed for *CRYAA*, *CRYAB*, *CRYBB2*, *CRYGA-D*, *CRYGS*, *GJA3*, *GJA8*, *LIM2*, and *MIP* (Table 1).

Blood samples (5–10 ml) were collected from the available family members; genomic DNA was isolated according to standard procedures [14]. The PCR conditions for genomic DNA as a template have been previously described in references [15-17]. Primers have been received from Utz Linzner (Helmholtz Center Munich, Institutes of Experimental Genetics and of Pathology, Neuherberg, Germany) or through commercial means (Invitrogen, Karlsruhe, Germany; MWG, Vaterstetten, Germany; and Sigma Genosys, Steinheim, Germany). Sequencing was done commercially (Sequiserve, Vaterstetten, Germany and GATC Biotech, Konstanz, Germany).

The mutation was confirmed by the presence or absence of a restriction site. Since the KORA (Cooperative Health Research in the Augsburg Region) Survey 2000 (S3) studied a population-based sample of 4,261 subjects aged 25–74 years during the years 1999–2001 [18], we could use this cohort as a population-based control. Ninety-six randomly chosen individuals of this cohort without cataracts were analyzed for the *GJA8* mutation.

For computer-assisted predictions, the proteomics tools of the ExPASy server were used.

## Results

### Clinical findings

**Figure 1:**
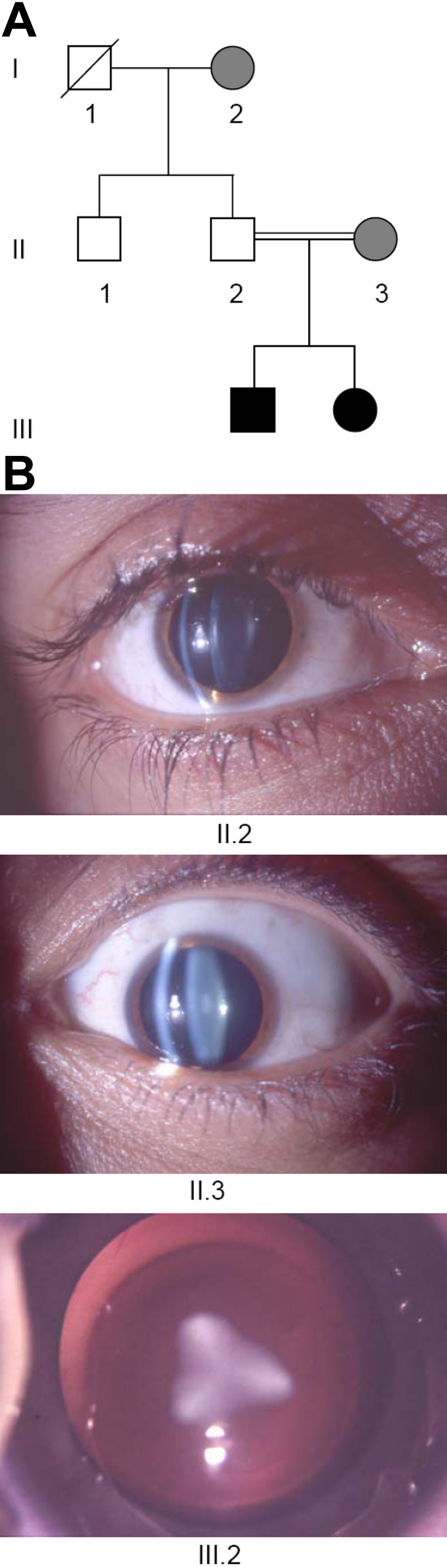
Cataracts in family D. **A**: Pedigree of family D indicates consanguinity in the second generation. The cataract appeared in the third generation and affects both children. The proband (III.2) is the younger daughter of the consanguineous parents. **B**: Ophthalmic details are given. On the left side, the healthy eye of the proband’s father (II.2) is given while on the right side, the slight embryonic nucleus opacity of her mother (II.3) is shown. Both were analyzed by a slit lamp. At the bottom, the triangular cataract of the proband (III.2) is seen immediately before the operation (using an operation microscope).

The proband, AD, is the youngest child of a three-generation family; her parents are consanguineous (cousins) from central Turkey (Figure 1A). Her mother and grandmother suffer from a discrete, symmetric opacity of the fetal lens nucleus; the opacity never limited their visual acuity. The proband’s father and uncle are without any lens opacity. The lenses of the proband were removed at the age of two weeks because of a dense, triangular nuclear cataract, which was symmetric in both eyes. At the periphery, additional opaque zones can be observed. The same clinical feature was observed in her older brother who was operated one year ago at the age of six weeks. A summary of the clinical findings in the family is given in Figure 1B.

### Molecular findings

**Figure 2:**
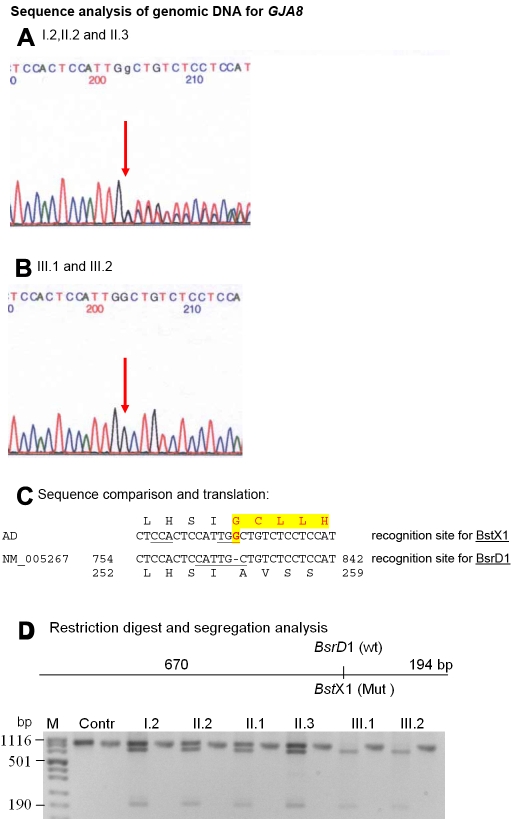
*GJA8* mutation in family D. Sequence analysis of *GJA8* genomic DNA indicates heterozygosity after position 776 (red arrow) for the proband’s parents, II.2 and II.3 (**A**) and a homozygous insertion of a “G” at this position in the cDNA from the proband’s lens mRNA (**B**). **C**: The comparison of the wild type sequence (NM_005267) with the proband’s sequence (AD) demonstrates that the insertion of the G after position 776 leads to an altered open reading frame. The new amino acid sequence is indicated by red letters in a yellow box. Moreover the mutation causes a loss of the restriction site for BsrD1 but creates a new one (BstX1). In the cDNA sequence, the A of the ATG start codon is counted as #1 and in the amino-acid sequence, the first Met is counted as #1. **D**: The restriction digest using BstX1 in the members of the family demonstrates that homozygosity of the mutation is present only in both severely affected children. The other family members are heterozygous, independent of the slight nuclear opacity. A control person from outside (Contr) is homozygous wild type, since BstX1 cannot cut.

For the molecular analysis, cDNA was obtained from the removed lens material. To understand the genetic basis of this cataract of spontaneous origin, we applied the functional candidate approach and analyzed the transcripts of 12 genes that had previously been shown to be involved in congenital cataract formation. Among them, we observed in the *GJA8* cDNA a homozygous insertion of one G after position 776 (c776insG) leading to a frame shift (Figure 2A-C). Protein translation programs predict a long open reading frame, which allows the translation of 123 novel amino acids.

The mutation creates a recognition site for BstX1 and destroys one for BsrD1 (Figure 2C,D); it was confirmed in the genomic DNA of the proband as well as in her brother. The parents, the grandmother, and the father’s brother are all heterozygous for this mutation (Figure 2D), independent of whether they had the slight nuclear opacity or not. Using the digestion schedule for BstX1 (present in the mutant), the mutation could not be detected in 96 healthy controls from Germany (data not shown).

## Discussion

Here, we describe the case of a child who was operated on for triangular cataracts within the first week of her life. Because there was no indication of any cataract-causing situation during pregnancy, a genetic analysis was performed to understand the etiology. The family history of cataracts was weak because only the mother and the grandmother had a slight opacity, which was obviously different from the cataract of the proband and her older brother. In this family, a recessive mode of inheritance was considered, since the parents are consanguineous. Since the size of the family did not allow genome-wide mapping, we applied a functional candidate approach testing for several well known cataract-causing genes (*CRYAA*, *CRYAB*, *CRYBB2*, *CRYGA*-*D*, *CRYGS*, *GJA3*, *GJA8*, *LIM2*, and *MIP*). Fortunately, we could make use of cDNA, which was available from the patient due to the sampling and immediate freezing of the operation material. The presence of the cDNA makes mutation screening faster and allows also the identification of splice variants.

In the proband, AD (as well as in her older brother), we observed a homozygous 1 bp insertion (c776insG) affecting the COOH-terminal part of connexin50. In both children, the homozygous mutation causes a triangular cataract, which was operated within the first weeks after birth. Since all family members who could be investigated were heterozygous for this mutation independent of a clear lens or slight lens opacity, this insertion causes a recessive mode of inheritance for the severe form. It remains an open question whether the slight opacities observed in the mother and grandmother are associated with this insertion. Since it was observed only in the female members of the family, it might be discussed as a sex-linked expressivity. Moreover, it might be due also to polymorphism(s) in other gene(s). However, the number of cases is too small to validate such speculations.

Since the 1 bp insertion changes the open reading frame and leads to a premature stop codon, the recessive nature of the mutation might be discussed as a result of a nonsense-mediated decay of the corresponding mRNA, and therefore, it is considered a functional null allele. However, this assumption might not be very likely because of three reasons:

1. DeRosa et al. [19] reported Cx50 being truncated after amino acid residue 290. The protein was found to be expressed and localized to the cell membrane in transfected HeLa cells. However, it also formed channels with a significantly impaired conductance.
2. The actual mutation was found in lens cDNA, which was obtained after lens surgery from a homozygous patient. Therefore, instability of this particular mRNA can be excluded, and the effect of the mutation needs to be explained on the basis of the impaired function of the COOH-terminal domain.
3. The cDNA is not contaminated by genomic DNA; therefore, an artificial amplification of genomic DNA from this cDNA can be excluded (data not shown).

Recently, another recessive insertion in the *GJA8* gene was reported from an Indian consanguineous family [9]. In contrast to the mutation described here, the insertion in the Indian family occurs after codon 203 and is predicted to result in the loss of the second extracellular domain and the subsequent transmembrane and cytoplasmic domains. Since our new insertional mutation leaves the four transmembrane domains unaffected, this particular mutation offers the possibility to analyze the function of this part of the protein in detail and independent of the transmembrane domains.

As mentioned above, most other *GJA8* mutations associated with cataracts affect the transmembrane domains or the extracellular loops and are characterized by a dominant mode of inheritance. These mutations include Arg23Thr [20], Val44Glu [21], Trp45Ser [22], Asp47Asn [23], Glu48Lys [24], Val79Leu [25], Pro88Ser [26], Pro88Gln [27], Pro189Leu [28], Arg198Glu [21], and Ser276Phe [29]. It is obvious from this list that most mutations detected so far affect the first half of the protein. In the mouse, one spontaneous mutation and two chemically induced cataract mutations have been reported, and all of them are dominant. They also affect the first transmembrane domain or the first extracellular loop (Gly22Arg [30], Asp47Ala [31], and Val64Ala [32]). The knockout of *Gja8* in the mouse leads to changes in the conductance only in homozygous mutants but not in heterozygotes [33], suggesting a recessive mode of inheritance (cataract data of heterozygous knockout mutants are not reported so far).

Besides the importance of *GJA8* for the transparency and functional integrity of the lens, *GJA8* is also expressed in the brain at least in the mouse. The Allen brain atlas demonstrates *Gja8* expression in the hippocampus, lateral ventricle, prefrontal cortex, amygdala, and cerebellum. Therefore, it is not surprising that *GJA8* is also considered to be associated with neurologic or psychiatric disorders like schizophrenia [34].

In conclusion, we demonstrated a homozygous 1 bp insertion within the *GJA8* gene in two children having congenital triangular cataracts. Since the heterozygous members of the consanguineous family show clear lenses or only very mild opacities without any effect on visual capability, the mutation is considered to be most likely causative for the recessive mode of inheritance. Since the mutation affects the COOH-terminal part of the Cx50 only, it might also be used to analyze the function of this particular domain in detail.
